# The Effects of Social Dance on Muscular Fitness and Longevity-Related Circulating Biomarkers in the Elderly

**DOI:** 10.3390/jfmk11030330

**Published:** 2026-08-24

**Authors:** Annamaria Mancini, Francesca Greco, Daniela Vitucci, Federico Quinzi, Maria Grazia Tarsitano, Loretta Francesca Cosco, Andreina Alfieri, Domenico Martone, Sara Dei, Rosa Ghirelli, Luca Gentile, Giulia Scalia, Pasqualina Buono, Gian Pietro Emerenziani

**Affiliations:** 1 Department of Medical, Human Movement and Wellbeing Sciences, Parthenope University of Naples, 80133 Naples, Italy; annamaria.mancini@uniparthenope.it (A.M.); lorettafrancesca.cosco@phds.uniparthenope.it (L.F.C.); andreina.alfieri@uniparthenope.it (A.A.); sara.dei@studenti.uniparthenope.it (S.D.); 2 CEINGE-Biotecnologie Avanzate “Franco Salvatore”, 80131 Napoli, Italy; daniela.vitucci@uniparthenope.it (D.V.); ghirelli@ceinge.unina.it (R.G.); scalia@ceinge.unina.it (G.S.); 3 Department of Experimental and Clinical Medicine, University of Magna Graecia, 88100 Catanzaro, Italy; f.greco@unicz.it (F.G.); fquinzi@unicz.it (F.Q.); emerenziani@unicz.it (G.P.E.); 4 Department of Mathematics and Computer Science, University of Calabria, 87036 Rende, Italy; 5 Department of Economics, Law, Cybersecurity and Sport Sciences, Parthenope University of Naples, 80133 Naples, Italy; domenico.martone@uniparthenope.it; 6 Department of Human Science and Promotion of Quality of Life, San Raffaele Open University of Rome, 00166 Rome, Italy; mariagrazia.tarsitano@uniroma5.it; 7 Department of Neurosciences, Biomedicine and Movement Sciences, University of Verona, 37129 Verona, Italy; 8 DaiMedLab and Transfusion Unit, AOU Università degli Studi di Napoli Federico II, 80131 Naples, Italy; luca.gentile@unina.it

**Keywords:** Social Dance, muscular fitness, Biological Antioxidant Potential (BAP), PASE, EQ-VAS

## Abstract

**Background**: Aging is characterized by functional decline in different organs and systems. The present study aimed to evaluate the effects of 6-month low-to moderate intensity supervised Social Dance (SD) program on muscular fitness, and clinical–biochemical and hematological parameters associated with healthy aging. Circulating CD34+ and serum antioxidant potential were assessed as exploratory outcomes. **Methods**: Seventy-nine elderly (aged > 65 years) were enrolled and randomized. Analyses included 49 participants who provided baseline and follow-up data and were assigned to the SD group (SDG, n = 24) or control group (CG, n = 25). Clinical–biochemical and hematological profiles, oxidative stress markers, circulating CD34+ cells, Physical fitness components and Physical Activity Scale for the Elderly (PASE) and EuroQol five-dimensional (EQ-5D) questionnaires were assessed at T0 and T1 to both groups. **Results**: Significant group × time interactions favored the SDG for HDL cholesterol (p=0.003), Hemoglobin (*p* = 0.031), and Hematocrit (*p* = 0.005). A significant increase in BAP (∆% = 33.3; *p* < 0.001) and PASE (∆%= 79.6 vs. 56.9; *p* < 0.05) were observed among the SDG; conversely, only a positive trend (∆% = 12.9 SDG vs. −5.1 CG) was observed for EQ-VAS at T1; these findings were not supported by significant group × time interaction. CD34+ cells increased significantly only in the CG. Isometric muscular performance declined in both groups. **Conclusions**: a 6-month SD program may provide beneficial effects on selected biochemical markers (i.e., a positive impact on HDL, hematological profile, antioxidant defenses) and perceived health outcomes in the elderly. However, it appears insufficient to preserve isometric muscular performance, suggesting that complementary resistance-based strategies may be needed to optimize neuromuscular adaptations.

## 1. Introduction

Ageing is the result of the accumulation of a large variety of molecular and cellular damage over time, leading to a gradual decline in physical, cognitive, and mental abilities and increased risk of disease [1]. In literature, elderly were classified into four subgroups: young elderly (64–74 years), elderly (75–84 years), very elderly (85–99 years), and centenarians [2]. A recent WHO report [1] highlighted that by 2050, the global population of people aged 60 and over will double, while the number of people aged 80 and over will triple between 2020 and 2050, reaching 426 million.

Healthy aging promotes better overall well-being, maintaining all intrinsic, functional, cognitive, and social abilities, thus preventing the onset of numerous non-communicable diseases [3].

A large body of evidence has demonstrated that structured Physical Activity (PA) promotes functional and motor adaptations across different stages of life [4], serving as a non-invasive therapy for improving physical and mental health, helping to prevent falls and chronic diseases, and preserving muscle fitness and metabolic health in the elderly population [5,6,7]. Further, sport training could contribute to the promotion of physical fitness and wellbeing in the elderly. Martone et al. [8] recently demonstrated that regular long-term football training improved bone mineral density in the legs and lower spine, increased lean and reduced fat mass, and improved cardiovascular health in older footballers. Other studies evidenced the effectiveness of exercise in improving metabolic parameters [9], cognitive function [10], and muscle fitness [11,12,13]. Therefore, together with other health-related components, the assessment of muscular fitness (i.e., muscle strength, endurance, and power) plays a fundamental role in the evaluation of age-related markers [14,15,16]. At the biological level, physical exercise can also influence skeletal muscle markers, circulating cellular and redox markers involved in tissue maintenance, and longevity [17,18]. Among these markers, circulating CD34+ progenitor cells and serum antioxidant capacity can provide complementary information on age-related regenerative potential and systemic redox homeostasis.

Current evidences also reveals that exercise mobilizes and enhances the function of stem cells (SCs), which play a crucial role in tissue repair, regeneration, and maintenance of physiological homeostasis [19]. Hematopoietic stem and progenitor cells (HSPCs) are characterized by the expression of specific cell surface markers such as CD34+. An increase in the circulating levels of CD34+ and CD34+/KDR+ subset cells was reported after a 30 min high/moderate-intensity running session or moderate-intensity cycling in healthy young adults; contrarily, a reduced effect was observed after different types of exercise in older compared to younger individuals [19,20,21]. It has been reported that the number of circulating CD34+ cells and their clonogenic capacity decrease with advancing age, while higher levels have been associated, in observational studies, with more favorable health and survival profiles in the very elderly [22,23]. These findings support the biological relevance of CD34+ cells as an indicator of age-related regenerative and vascular homeostasis, although their circulating concentration should not be considered a validated clinical surrogate marker. The available evidence therefore does not establish whether repeated low- to moderate-intensity exercise can alter resting levels of circulating CD34+ cells in the elderly [24]. No evidence has been provided on the effect of SD in circulating CD34+ cell levels.

Furthermore, increasing evidence suggests that aging and age-related diseases are correlated with increased serum inflammation and oxidative stress (OS), which are characterized by an imbalance between Reactive Oxygen Species (ROS) production and serum Biological Antioxidant Potential (BAP); this phenomenon is most evident in the elderly [25].

Although exercise temporarily increases the production of ROS, repeated and appropriately dosed PA can induce adaptive responses that strengthen endogenous antioxidant defenses. In the elderly, aerobic training has been shown to partially restore the activation of Nrf2-related antioxidant signaling, which is impaired with advancing age [26]. However, the extent of this adaptation depends on the type and intensity of exercise, baseline fitness level, and the oxidative or antioxidant biomarker being examined [27]. However, since this response has not previously been established for social dancing, serum antioxidant potential was also considered an exploratory biomarker outcome. Innovative activities like dance, tai chi, and yoga are recently increased among older population. Compared to traditional exercise sessions, dance results in a multi-component activity, being more engaging and attractive [28]. Recently, it has been demonstrated that regular engagement in a dance group enhances lower limb muscle strength, balance, and mental wellbeing in the elderly [29]. It also improves the management of chronic diseases like cardiovascular disease, cognitive function, and general well-being and reduces frailty in individuals with mild cognitive decline [30,31].

Nevertheless, previous studies on dance have focused primarily on balance, mobility, cognitive performance, psychological well-being, and quality of life. The available studies are also characterized by considerable heterogeneity in terms of dance style, intensity and duration of the intervention, control conditions, and selection of outcomes [32]. Consequently, their results provide limited information on the effects of a standardized, long-term Social Dance intervention on muscular fitness and circulating biomarkers in the elderly.

The Social Dance (SD) represents a new paradigm of recreational dance, founded on popular music belonging from the five continents that include low-intensity choreography. Only few research has been conducted so far on the effects mediated by SD on behavior of elderly [28,33].

No evidence was provided to date on the effects of SD programs on muscular fitness and circulating biomarkers associated with active and healthy ageing. Therefore, the present study aimed to evaluate the effects of a 6-month low-to-moderate intensity supervised SD program on biometric, clinical–biochemical, and hematological parameters, muscular fitness components, and antioxidant serum potential associated with active and healthy aging in the elderly.

We hypothesized that, compared to the control group, participation in the 6-month SD program would lead to greater improvements in the study’s main outcomes, namely muscular fitness and hematological, clinical, and biochemical parameters associated with healthy aging in the elderly; as exploratory outcomes, we hypothesized that the intervention would increase levels of circulating CD34+ cells and enhance serum antioxidant potential.

## 2. Materials and Methods

### 2.1. Study Design

**Figure 1:**
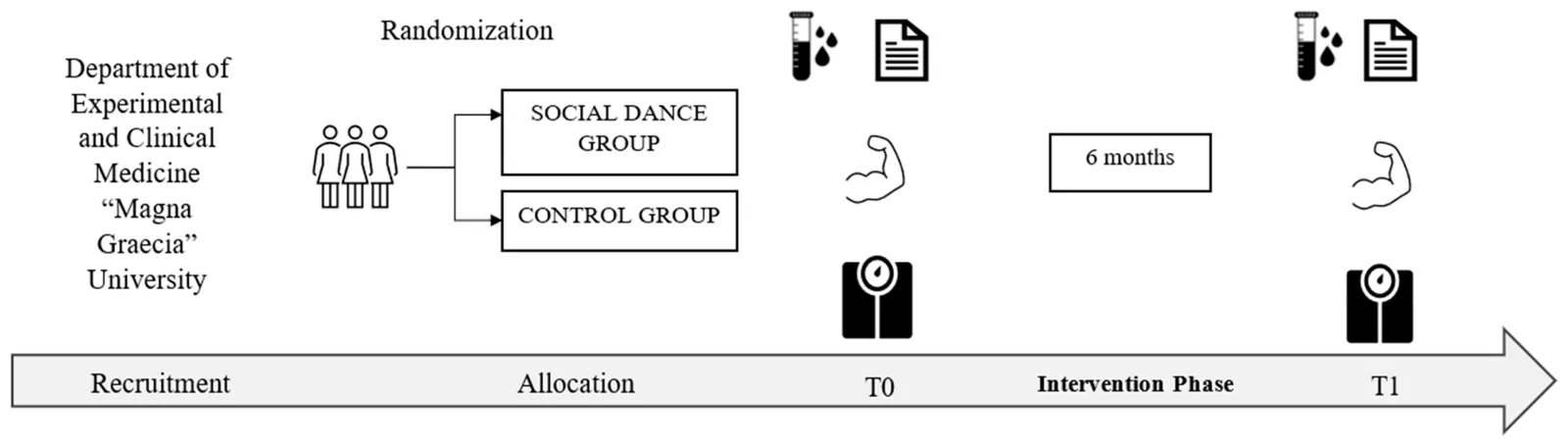
Study design flowchart. T0, baseline; T1, after 6-month of SD program intervention.

This study adopted a randomized control trial design. Volunteers were enrolled at the Department of Experimental and Clinical Medicine of Magna Graecia University, Catanzaro, Italy. All participants were evaluated at the Physical Exercise and Sports Science laboratory of “Magna Graecia” University of Catanzaro, whereas biochemical analysis was performed at the Parthenope University of Naples. At the baseline assessment (T0), participants were randomly assigned to a SD group (SDG) or no-intervention (control group, CG). The SD program was performed at dance schools “Non solo Liscio” in Catanzaro and “UISP danza Calabria” in Tortora Marina (CS). All the volunteers were recommended an active lifestyle, further, the SDG performed a 6-month low-to-moderate SD program (two times/week), while the CG were not involved in any structured physical exercise. The study design adopted is reported in Figure 1. All experimental procedures detailed in the following paragraphs comply with the Declaration of Helsinki. After being informed of the research procedures, goals, and risks and the benefits of participating in this study, the participants signed a written informed consent form approved by the ethical committee of Calabria Region (n.12/2023).

### 2.2. Participants

A total of 79 elderly of both genders were recruited and enrolled in the study. Participants were randomly assigned (1:1) to the SDG or CG using a computer-generated random allocation sequence created in Microsoft Excel. To ensure allocation concealment, group assignments were revealed sequentially from a password-protected file by an independent researcher and were disclosed only after participant enrollment and completion of the baseline assessment. 

Owing to the nature of the intervention, participants and instructors could not be blinded. During the 6-month intervention, 30 participants withdrew, mainly for personal reasons, including unavailability for the follow-up assessment. Of these, 49 participants completed the experimental procedure (SDG, n = 24; CG, n = 25). Participants’ flow diagram is reported in Figure S1.

Inclusion criteria included age > 65 years old. Exclusion criteria include recent major cardiovascular events (including stroke and myocardial infarction), unstable angina, heart failure (NYHA III-IV), and respiratory failure, during the last 6-month period; neoplastic or autoimmune diseases and severe cognitive impairments; individuals taking coumarin drugs and women taking hormone replacement therapy; or any contraindications to the practice of PA assessed with the Physical Activity Readiness Questionnaire (PAR-Q+) [34]. Participants’ characteristics at baseline (T0) and after the intervention phase (T1) are reported in Table 1.

### 2.3. Experimental Protocol

The SDG performed supervised SD lessons that lasted approximately 2 h (two times/week) and consisted of different choreographies, which included rhythmic and simple movements, typically of Latin dance (Cha Cha Cha, Rumba, Jive, Tango, and Merengue), Caribbean dance (Salsa and Bachata), and folkloristic dance (Tarantella and Pizzica) [28]. In all the described dances, the leaders will be the men, and the followers will be the women. Each dance class was supervised by an expert dance teacher and included movements of SD (also resembling activity of daily living and strengthening exercises) performed at a beginner/intermediate level. The dance class was composed by warming up at the beginning with low intensity, followed by moderate intensity, and ending with cool-down. The exercise intensity was monitored with the Rate of Perceived Exertion (RPE) scale using the CR-10 scale weekly [35]. The CG maintained its lifestyle routine and was not involved in any structured physical exercise, but they were told to have an active lifestyle. Both groups received nutritional counselling to follow the guidelines of the Mediterranean diet. Attendance rate for the SDG was calculated based on participation in the supervised dance sessions. Since no structured intervention was provided to the CG, adherence to an intervention protocol was not applicable. Compliance with the study procedures in the CG was assessed based on completion of the scheduled assessments.

### 2.4. Physical Fitness Evaluation

At baseline (T0) and after 6 months of intervention (T1), participants underwent a physical fitness examination which involved the following measurements:

Height was measured barefoot to the nearest 0.1 cm using a stadiometer (SECA, Intermed S.r.l., Milano, Italy). Participants stood with their body mass evenly distributed on both feet, heels together, and head positioned in a midline position (Frankfort horizontal plane), with their arms hanging loosely.

Body mass and body composition were measured using a bioelectrical impedance method (BIA ACCUNIQ 360, Daejeon, Republic of Korea) while participants wore minimal clothing and removed any metal accessories before the analysis was conducted. After entering the participant’s height, date of birth, and gender into the device, their body mass was automatically measured. During the measurement, participants stood in an upright position, with arms at about a 30° angle away from the trunk. This position was maintained until the end of the measurement, and no talking or movement was allowed. The variables of interest were the body mass index [BMI = body mass (kg)/height^2^ (m)], total body water (TBW), the fat mass (FM) expressed as a percentage of body mass, and skeletal muscle mass (SMM) expressed in kilograms.

Cardiorespiratory endurance was evaluated using the 2 min Step Test (2mST) [36]. Participants were asked to step while lifting each knee to a point halfway between the kneecap and the iliac crest for 2 min. The number of complete steps performed correctly was recorded.

Lower body maximal isometric peak torque (PeakT) was assessed using a force sensor (MuscleLabTM 6000, Porsgrunn, Norway) on a leg extension of the dominant lower limb as described elsewhere [30]. Participants were instructed to contract muscle quadriceps as hard and fast as possible for 3 s. The rate of torque development (RTD) as a measure of explosive strength (i.e., muscle power) was also analyzed.

Lower body muscular endurance (Fmean) was assessed as following: the participant was seated as in the maximal isometric leg strength assessment, and the test consists of 12 isometric maximal contractions lasting 3 s with a rest of 5 s between repetitions. Endurance was assessed through the mean force parameter, computing the mean forces of the twelve contractions for each participant. Moreover, a fatigue index was calculated as follows [37]: [(maximal force at 1st repetition−maximal force at 12th repetition)/maximal force at 1st repetition] × 100).

The chair stand test (CST) evaluated lower limb muscular endurance. Participants were asked to stand up and sit down ten consecutive times on a standard chair without armrests in the shortest possible time. The time (s) to execute the task was recorded with a stopwatch and used for the analysis [38].

Specific power assessment was performed as follows: the specific power was calculated by dividing the muscle power by the skeletal muscle mass (muscle power (W)/SMM (kg)). Muscle power was estimated using Takai’s equation: muscle power (W) = (L − 0.4) × body mass × g × 10/T. In this equation, L represents the participant’s limb length (in meters), measured from the greater trochanter of the femur to the lateral malleolus. The value 0.4 corresponds to the height of the chair (in meters), g is the acceleration due to gravity (9.8 m·s^−2^), and T is the time (in seconds) taken to complete the test [38].

### 2.5. Questionnaires

The Physical Activity Scale for the Elderly (PASE) [31] was administered to measure the level of self-reported PA in individuals using the Italian validated version [39]. The questionnaire is composed of items regarding occupational, household, and leisure activities during the previous 7-day period. The score obtained from leisure and recreational activities, household activities, and overall score was used for the analysis.

The EuroQol five-dimensional (EQ-5D) instrument was administered using the Italian version [40]. The EQ-5D questionnaire consists of two parts. The first part is a descriptive system that contains five dimensions; the second part is the EuroQol visual analogue scale (EQ-VAS), which is a 0–100 scale where respondents indicate their overall health status. EQ-VAS results were used for the analysis.

### 2.6. Blood Sampling and Analysis

Following an overnight fast, peripheral blood and serum samples were collected from each participant at the clinical center in Catanzaro (Italy) at baseline (T0) and after the 6-month intervention period (T1). Blood samples were collected via venipuncture into evacuated tubes containing appropriate anticoagulants (e.g., EDTA for hematological analysis and CD34+ cell quantification). All samples were immediately transported to the laboratory at the CEINGE Biotecnologie Avanzate in Naples, under controlled temperature conditions (+4 °C) for processing and analysis. For serum separation, whole blood was centrifugated at 2000× *g* for 10 min at 4 °C. The resulting serum was aliquoted into 500 µL volumes and immediately stored at −80 °C to preserve analyte stability until the analysis of antioxidant parameters. Biochemical and clinical parameters—including fasting blood glucose, total cholesterol, LDL, HDL, triglycerides, creatinine, AST, and ALT—were measured in the serum aliquots. Hematological profiles and CD34+ hematopoietic stem cell counts were evaluated using fresh whole blood samples.

### 2.7. CD34+ Cells Evaluation

A 1 mL aliquot of EDTA-anticoagulated whole blood was lysed using Pharm Lyse™ solution (BD Biosciences) for 20 min at room temperature in the dark. Following centrifugation, the cell pellet was washed twice with phosphate-buffered saline (PBS). The cells were then incubated at 4 °C for 15 min with fluorochrome-conjugated monoclonal antibodies against CD34 and CD45 [41]. After incubation, the samples underwent a final wash step prior to flow cytometric acquisition.

Data acquisition was performed using a DxFlex flow cytometer (Beckman Coulter Life Sciences, Brea, CA, USA), and subsequent data analysis was conducted with CytExpert software v2.5 (Beckman Coulter). The following antigenic markers were evaluated: CD45-FITC and CD34-PE.

A total of 500,000 events were acquired per sample. Cell doublets were systematically excluded using FSC-A vs. FSC-H dot plots. Leukocyte populations were identified via CD45 vs. SSC-A gating, while CD34+ cells were characterized using CD34 vs. SSC-A dot plots. All results were expressed as percentages.

### 2.8. BAP Test and d-ROMs Test

Both tests were performed on serum. Sera collected and stored at −80 °C were thawed in ice and analyzed for the Biological Antioxidant Potential (BAP,cod. MC436, DIACRON International S.r.l., Grosseto, Italy) and for derivates of Reactive Oxygen Metabolites (d-ROMs, DIACRON s.r.l. cod. MC001) tests, according to the manufacturer’s data sheet.

The d-ROMs test quantifies the ROMs (primarily hydroperoxides, ROOH) present in the biological sample. The test utilizes the principle of a Fenton-type reaction: briefly, in the presence of iron (released from plasma proteins by an acidic buffer, R2 reagent), the hydroperoxides react to generate highly reactive free radicals, namely alkoxyl (R-O^) and peroxyl (R-OO^) radicals. These radicals then react with a substituted aromatic amine (A-NH2 contained in a chromogenic mixture, reagent R1), leading to the oxidation of the amine, resulting in the formation of a stable, pink-colored derivative directly proportional to the number of ROMs originally present in the sample. On the other hand, the BAP Test assesses the total antioxidant capacity of serum. Antioxidants present in serum (the reducing system) reduce the ferric ions (Fe3+) to ferrous ions (Fe2+). This reduction causes a change in the chromogen complex, resulting in a measurable decolorization of the solution. The extent of decolorization is directly proportional to the antioxidant potential of the sample analyzed [42].

### 2.9. Statistical Analysis

A statistical analysis was conducted using IBM^®^ SPSS Statistics software version 23.0 (SPSS Inc., Chicago, IL, USA) and Jamovi software (Version 2.3.26.0). An a priori sample size estimation performed using G-Power 3.1.9.2 showed that 24 participants per group were sufficient to achieve a statistical power of 1 − β = 0.80 (effect size: 0.3; α = 0.05) based on previous literature [32,43]. All analyses were conducted using a complete-case analysis, including only participants with complete baseline and follow-up data.

The Shapiro–Wilk statistical test was used to check the normality of the distributions. The variables that had a non-parametric distribution were expressed as medians and interquartile range, while those that had normal distribution were expressed as mean and standard deviation. Two-way (group × time) ANOVA for repeated measures on time was used to detect the significant effects of two main factors: group (SDG vs. CG) and time (T0 vs. T1) followed by Bonferroni or paired-*t* test post hoc analysis where appropriate. Although Bonferroni correction was applied for post hoc comparisons, no additional adjustment was performed across the different outcome measures. Therefore, secondary outcomes were interpreted with caution.

Assumptions of sphericity and homogeneity of variance were evaluated using Mauchly’s test and Levene’s test, respectively. Since both assumptions were satisfied (*p* > 0.05), standard uncorrected degrees of freedom were reported. A paired samples *t*-test was performed to evaluate changes from baseline to post-intervention, while the Wilcoxon signed-rank test was used for non-normally distributed paired data. Linear regression analysis was used to assess the influence of gender on the dependent variables. The similar baseline characteristics of the two groups were verified using an unpaired *t*-test at T0. Analysis of Covariance (ANCOVA) with baseline age as a continuous covariate was used to adjust for baseline age differences. For all analysis, the level of significance was set at *p* < 0.05. Data are expressed as percentage differences (∆%) relative to the baseline (T0), calculated as the relative increase or decrease over the initial value: ∆% = [(T1 − T0)/T0] × 100.

## 3. Results

A total of 33% of the participants in the overall sample were taking antihypertensive medications, while 14% were on statin therapy. In the CG, 48% of participants engaged in walking as a leisure and recreational activity, whereas the remaining participants reported participating in other recreational and household activities. Attendance at the supervised dance sessions in the SDG was 62%, while completion of the scheduled follow-up assessments in the CG was 60%. Drop-out was primarily due to personal reasons, including unavailability for the follow-up assessment. No withdrawals were attributed to adverse events related to the intervention. In the SDG, RPE (CR-10) values ranged between 2–5 (average: 2.7 ± 0.9).

Participants’ biometric data, biochemical data, and hematological profiles are reported in Table 1, Table 2, and Table 3, respectively. Regarding baseline characteristics (Table 1), SDG participants were slightly older than those in the CG (74.8 ± 6.3 vs. 70.1 ± 7.3 years, respectively), while height was comparable between groups (1.61 ± 0.09 for CG and 1.60 ± 0.09 m for SDG). Diastolic Blood Pressure (DBP) decreased significantly in the CG total sample (*p* < 0.001). Significant drops were observed in both males (*p* < 0.001) and females (*p* < 0.05). No statistically significant changes were observed for the total SDG sample or individual subgroups from T0 to T1. To control age differences between groups, baseline DBP levels were evaluated using ANCOVA with age as a covariate. At T0, no significant difference was found between groups (F_1,46_ = 2.97, *p* = 0.092, η^2^ = 0.058), and age did not act as a significant covariate (F_1,46_ = 2.63, *p* = 0.112, η^2^ = 0.051), confirming equivalent baseline blood pressure profiles prior to the intervention. The CG total group showed a significant reduction to a median value of Systolic Blood Pressure (SBP) of 76.0 mmHg (*p* < 0.001). Both males and females experienced a significant decrease (*p* < 0.05). Statistically significant reduction was found in the female subgroup (*p* < 0.05) and in the total SDG sample (*p* < 0.05), reaching a median of 75.5 mmHg. Similarly, no significant group difference was found for baseline level of SBP when controlling for age (F_1,46_ = 1.85, *p* = 0.181, η^2^ = 0.036). Moreover, Heart Rate (HR) dropped significantly from 78.6 ± 13.0 bpm to 72.5 ± 8.8 bpm (*p* < 0.01), with significant reductions in both subgroups (*p* < 0.05). HR values remained stable in the SDG elderly from T0 to T1 with no statistically significant variations (Table 1). When evaluating baseline HR at T0, ANCOVA revealed a significant main effect of group (F_1,46_ = 8.85, *p* = 0.005, η^2^ = 0.161), indicating a baseline difference between groups, whereas age did not act as a significant covariate (F_1,46_ = 0.02, *p* = 0.883, η^2^ = 0.000).

In terms of metabolic and lipid profiles, a highly significant improvement was observed exclusively in the SDG regarding HDL cholesterol levels from T0 to T1. Specifically, significant group × time interactions were observed for serum HDL cholesterol (F_1,47_ = 9.65, *p* = 0.003, η^2^ = 0.011), with the SDG elderly showing a significant increase in HDL (median from 50.5 to 53.3 mg/dL, *p* < 0.001), driven by significant improvements in both males (45.7 ± 7.0 to 52.5 ± 9.7 mg/dL, *p* < 0.01) and females (*p* < 0.05). Conversely, HDL levels remained unchanged in the CG (Table 2). To test the age difference, baseline HDL levels were evaluated using ANCOVA with age as a covariate: no significant differences between groups at T0 (F_1,46_ = 0.05, *p* = 0.827, η^2^ = 0.001) were found, further, age did not act as a significant covariate (F_1,46_ = 0.06, *p* = 0.812, η^2^ = 0.001), confirming equivalent baseline lipid profiles prior to the intervention.

An evaluation of hematological parameters from T0 to T1 revealed positive adaptations in the oxygen-transport capacity primarily within the SDG. In particular, significant group × time interactions were observed for serum Hb (F_1,47_ = 4.93, *p* = 0.031, η^2^ = 0.004), as well as for Hct (F_1,47_ = 8.57, *p* = 0.005, η^2^ = 0.011); further, a highly significant increase of Hb levels (rising from 13.5 ± 1.5 to 14.0 ± 1.9 g/dL; *p* < 0.001), was observed in the SDG elderly at T1. On the contrary, no significant variations were observed for Hb levels in the CG group, despite a little increase detected exclusively in CG males (14.7 ± 1.3 to 14.9 ± 1.2 g/dL, *p* < 0.05). Hematocrit (Hct) and Red Blood Cells (RBCs) mirrored the Hb trends, driven by significant increase in the SDG elderly (*p* < 0.01). No statistically significant modifications in Hct and RBCs were found in the CG total group or its respective subgroups (Table 3). At baseline (T0), no significant main effect of group was observed for any hematological variable, including Hb (F_1,46_ = 1.38, *p* = 0.246, η^2^ = 0.027), Hct (F_1,46_ = 1.76, *p* = 0.191, η^2^ = 0.034), RBCs (F_1,46_ = 0.13, *p* = 0.717, η^2^ = 0.003), and WBCs (F_1,46_ = 0.59, *p* = 0.445, η^2^ = 0.013). Although age was significantly associated with baseline Hct (F_1,46_ = 4.70, *p* = 0.035, η^2^ = 0.090), controlling for age confirmed that both groups had equivalent baseline hematological profiles prior to the intervention.

To evaluate the potential influence of gender on the observed outcomes, a linear regression analysis was performed. Notably, the interaction term for gender was not statistically significant (*p* > 0.05) indicating that gender does not significantly moderate the effect of the primary predictors (Table S1).

As shown in Table 4, CD34^+^ circulating cells increased in both groups, but significantly only in the CG (*p* < 0.05); no significant interaction between time × group (F_1,47_ = 0.22, *p* = 0.644, η^2^ = 0.001) was observed. In order to evaluate if SD protocol gave an effect on the serum biological antioxidant power, we performed the BAP test assay: no significant interaction between time × group (F_1,47_ = 1.35, *p* = 0.239, η^2^ = 0.008), and a significant main effect of time on BAP (F_1,47_ = 23.91, *p* < 0.001, η^2^ = 0.138) was found. Bonferroni post hoc analysis revealed a significant effect in BAP between T0 and T1 in the SDG group (*p* < 0.001). Analyzing the Reactive Oxygen Metabolites (free radical derivatives) in the participants’ serum, we found no significant time × group interaction effect (F_1,47_ = 0.06, *p* = 0.816, η^2^ = 0.000) or main effect of time (F_1,47_ = 0.01, *p* = 0.907, η^2^ = 0.000). Indeed, on average, both the CG and the SDG exhibited elevated levels of oxidative stress (401–500 UCARR). To control age disparity between groups, baseline (T0) profiles were evaluated using ANCOVA with age as a covariate. At T0, no significant group differences were observed for BAP (F_1,46_ = 0.11, *p* = 0.742, η^2^ = 0.002), d-ROMs (F_1,46_ = 0.06, *p* = 0.816, η^2^ = 0.001), or CD34+ cell number (F_1,46_ = 0.01, *p* = 0.905, η^2^ = 0.000). Furthermore, age did not act as a significant covariate for any of these markers (*p* = 0.980, *p* = 0.448, and *p* = 0.331, respectively), confirming equivalent pre-intervention antioxidant, oxidative stress, and endothelial progenitor cell profiles across groups.

Participants’ results on physical fitness and questionnaires are reported in Table 5. To evaluate whether the age gap influenced baseline parameters, T0 values were analyzed using ANCOVA with age as a covariate. At T0, no significant group differences were found for body composition (SMM: F_1,46_ = 0.11, *p* = 0.745, η^2^ = 0.002), physical performance outcomes (2MST: F_1,46_ = 0.52, *p* = 0.474, η^2^ = 0.010; PeakT: F_1,46_ = 0.01, *p* = 0.980, η^2^ = 0.000; RTD: F_1,46_ = 0.21, *p* = 0.649, η^2^ = 0.004; Fmean: F_1,46_ = 0.02, *p* = 0.889, η^2^ = 0.000), or self-reported PA and well-being status (PASE total: F_1,46_ = 2.96, *p* = 0.092, η^2^ = 0.060; EQ-VAS: F_1,46_ < 0.01, *p* = 0.973, η^2^ = 0.000; fatigue index: F_1,46_ = 3.71, *p* = 0.060, η^2^ = 0.074). Age did not act as a significant covariate for the majority of baseline parameters (all *p* > 0.05) analyzed, with the exception of EQ-VAS (F_1,46_ = 4.81, *p* = 0.033, η^2^ = 0.095). Nevertheless, adjusting for age we confirmed equivalent SMM, physical performance, and self-reported PA and well-being status at baseline across groups prior to the intervention.

When evaluating time effects via ANOVA, a significant main effect of time on SMM (F_1,47_ = 6.942, *p* < 0.05, η^2^ = 0.129), 2MST (F_1,47_ = 4.980, *p* < 0.05, η^2^ = 0.096), PeakT (F_1,47_ = 14.184, *p* < 0.05, η^2^ = 0.232), RTD (F_1,47_ = 18.8491, *p* < 0.01, η^2^ = 0.287), Fmean (F_1,47_ = 6.784, *p* < 0.05, η^2^ = 0.126), PASE leisure activities (F_1,47_ = 14.202, *p* < 0.01, η^2^ = 0.232), PASE household activities (F_1,47_ = 4.031, *p* = 0.05, η^2^ = 0.079), and PASE total score (F_1,47_ = 6.784, *p* < 0.01, η^2^ = 0.211) was found. Post hoc analysis showed that SMM and 2MST are reduced in both groups at T1 compared to T0, with the lowest level in the CG (*p* < 0.05); on the contrary, PeakT, Fmean, and RTD are lower in the SDG at T1 vs. T0 (*p* < 0.05). PASE (leisure, household, total) scores were significantly higher at T1 than at T0 in both groups with greater scores in the SDG group (*p* < 0.01) indicating a greater quality of perceived fitness and self-reported health status in the SDG (Table 5).

Although no significant main effect of time on the other variables of interest (i.e., TBW, fatigue index (FI), EQ-VAS were found, it is interesting to note a greater increase in FI in the SDG at T1 compared to T0; moreover, we found a slight reduction trend in FM percentage in the SDG at T1; similarly, the increase in EQ-VAS score (about 13%) in the SDG at T1 compared to the reduction (−5.09%) in the CG indicates a reduction in overall health status perceived in the CG.

## 4. Discussion

The present study evaluated the impact of a 6-month supervised low-to-moderate SD program on key dimensions of healthy aging in the elderly. In particular, we mainly focused on physical fitness, hematological, and clinical–biochemical parameters associated to healthy aging; circulating CD34+ cells and serum BAP and d-ROMs were also analyzed as exploratory indicators of regenerative and antioxidant status. Further, self-reported PA and well-being status were also assessed to provide a broader perspective on the potential effects of the SD intervention. Sedentary lifestyle is commonly observed among elderly: physical limitations, time constraints, and culture represent the main determinants of this unhealthy behavior. Dancing represents an attractive form of PA that can be tailored to fit a large older population. SD, which requires a low-to-moderate intensity engagement, not aimed at performance or competition, and promotes socialization and movement through traditional dance choreography is a useful tool to promote physical and mental well-being in older adults [28,44]. Previous studies have reported beneficial effects of dance-based intervention on physical fitness and well-being in elderly people [45,46]. However, the effects of dance interventions vary widely across different dance styles and depend on the duration and characteristics of the training program [32,47]. Indeed, age-related reductions in muscle mass and strength typically progress in the elderly, in particular in sedentary individuals [17,48,49]. Isometric muscular performance (i.e., PeakT, RTD, and Fmean) decreased in both groups over the 6-month intervention period, with larger numerical reductions observed in the SDG than in the CG. One possible explanation is that nearly half of the participants in the CG reported engaging in regular self-directed brisk walking as part of their habitual lifestyle. Although walking is not specifically designed to improve maximal isometric force, regular weight-bearing locomotor activity may have contributed to maintaining lower-limb neuromuscular function and may partly explain the lower decline in isometric strength parameters observed in the CG. Nevertheless, walking habits were self-reported but not objectively quantified; therefore, this interpretation should be considered speculative and warrants confirmation in future studies. Although not statistically significant, the greater relative increase in FI% observed in the SDG compared with the CG paralleled the descriptive reductions in PeakT, RTD, and Fmean, suggesting a possible but unconfirmed pattern of neuromuscular adaptation. However, this observation should be interpreted with caution because the SDG presented lower baseline FI values than the CG, and no significant between-group differences were detected. Additionally, regression analysis revealed no significant gender × group interactions across all evaluated parameters (Table S1), indicating that gender did not influence intervention outcomes.

Our findings are in contrast with those reported by Vaccaro et al. where improvements in physical fitness among older adults following a SD-based intervention have been found [28]. These differences could be ascribed to the differences in exercise intensity, structure, and overall training volume. Indeed, in the current study, the SD intervention was performed only two times per week and was proposed as recreational SD. Therefore, compared with a control group mainly engaged in activities like walking and maintaining an active lifestyle, the SD program did not appear sufficient in improving muscular fitness outcomes, particularly those related to isometric strength performance. Furthermore, the variety of dance styles included in the intervention may not have provided a sufficiently specific neuromuscular stimulus to effectively enhance muscular strength-related adaptations. Nevertheless, recent evidence suggests that recreational and socially engaging dance interventions may still represent a feasible strategy to support active aging, well-being, and functional health in the elderly, even when not specifically designed as structured strength and balance training programs [50,51]. Indeed, despite the lack of improvement reported in middle-aged ballroom dancers by Bonavolontà et al. [43], the present findings did not provide evidence that SD slowed the decline in muscular fitness compared with the control condition. The observed changes in muscular fitness should therefore be interpreted cautiously, whereas the improvement in self-reported PASE score may represent a potentially relevant secondary finding. The participation rate in the intervention group was moderate, which may have influenced the magnitude of the adaptations, particularly the muscular fitness outcomes that are closely related to training exposure.

It is important to underline that, since no significant group × time interaction was detected for the isometric outcomes, the results cannot support an effect of SD intervention on preservation, improvement, or slowdown of the decline in muscle capacity compared to the CG; further, the models related to FI% and PASE are purely descriptive and serve only to formulate hypotheses.

Previous reports evidence that dance therapy reduces Systolic and Diastolic Blood Pressure and improves HDL lipoprotein serum concentration in the elderly, contributing to the prevention or delay of atherosclerosis and cardiovascular disease in this population [52,53]. Further, aerobic exercise could reduce 5–10 mmHg DPB, or 1–6 mmHg SBP in hypertensive patients. It is relevant to observe that a reduction in blood pressure of 5 mmHg correspond to a 13% decrease in stroke risk [54,55]. Here, we evidence a significant reduction in SBP in both the CG and SDG (about 6 mmHg and 5 mmHg respectively) and a reduction trend in DBP and HR in the SDG at the end of the intervention. These results may be ascribed to the relatively low-to-medium intensity of SD program and to the antihypertensive drugs used by the 33% of participants.

Long-term aerobic exercise improves fat utilization for energy in the skeletal muscle and adipose tissue, leading to a reduction of blood lipid levels in middle-aged and elderly population [56]. Further, lifelong football training, which includes an aerobic component, positively improves the lipid profile in elderly players [17]. Dance therapy, induce an improvement in serum lipoprotein asset with HDL lipoprotein increase [52].

In the elderly, improvement in HDL lipoprotein is often challenging. We observe a significant increase in HDL lipoprotein serum levels in the SDG at the end of the intervention. These results provide further support to the limited available evidence on SD programs and metabolic health [57]. Our results reinforce the previous evidence, demonstrating that the program of 6-months of supervised SD low-to-moderate intensity seems to be sufficient to induce lipid remodeling and a slight reduction in FM% in the SDG elderly, contributing to the reduction in cardiovascular risk [58,59].

Aging is characterized by a decline in endogenous antioxidant defenses [60,61]. Several studies have evidenced that regular exercise programs have beneficial effects on the balance between serum oxidants increase and enhancement of antioxidant defense mechanisms [62]. Specifically, serum BAP scores indicate the overall biological capacity to counteract oxidative stress [63].

In the present study, both the SDG and CG have elevated concentrations of oxidative sera stress markers (d-ROMs) compared to reference intervals for elderly [25]. On the contrary, 6 months of the SD program were sufficient to increase serum BAP activity predominantly in the SDG compared to the CG (+33.3% vs. 19.1%) aligning to hermetic stimulus evidenced in Radak et al. [64]. As an exploratory endpoint, the increase in serum BAP activity in the SDG suggests a potential improvement in biological capacity to counteract oxidative stress. However, as exploratory outcomes, these biological adaptations should be considered preliminary [62,65]. Our data, for the first time, also evidence a positive effect on hematological profile in the SDG with an increase in Hb concentration and Hct% at the end of the intervention. Aging is associated with a reduction in the circulating number and functional capacity of hematopoietic stem and progenitor cells, including CD34+ cells [48,49,66]. Age-related alterations in HSC function contribute to immunosenescence and have been associated with increased susceptibility to hematologic malignancies and vascular dysfunction in the elderly [67].

Active lifestyle and exercise positively modulate the expression and activation of hematopoietic stem cells (HSCs) and circulating CD34+ progenitor cells [67,68,69]. However, most of the available evidence pertains to the transient mobilization of these cells following acute, moderate- to vigorous-intensity exercise, particularly in young or physically fit individuals. In contrast, the effects of repeated exercise interventions on resting CD34+ cell expression remain inconsistent, in particular in the elderly. Stress reduction and active lifestyle also positively influence HSC cells function and improve mental and physical health in the elderly [70,71], although evidence of a direct effect of stress reduction on circulating CD34+ cell levels remains limited. We found a significant increase in antioxidant activity (BAP) only in the SDG, associated with a slight increase in CD34+ circulating cells. Although the SDG showed an upward trend, a significant increase in CD34+ cells was unexpectedly observed only in the CG. In fact, the observed increase in the CG may reflect biological and interindividual variability in circulating CD34+ cells. Furthermore, exercise-induced adaptations may result in transient cell mobilization or changes in progenitor cell function, homing, or clonogenic capacity without producing a sustained increase in the number of resting CD34+ cells.

Therefore, it is not yet clear whether the SD intervention has a direct impact on the mobilization of CD34+ cells, and our results should be considered preliminary. Nevertheless, to our knowledge, this study provides the first preliminary evidence that a SD program may improve serum antioxidant activity in elderly people.

## 5. Conclusions

In conclusion, 6 months of a supervised SD program yielded mixed results, highlighting both the potential and the boundaries of this intervention in the elderly. Unlike standard exercise prescriptions that often suffer from low adherence, SD leverages enjoyment and social interaction to maintain participation. However, the SD intervention resulted insufficient to preserve or improve the muscular isometric efficiency suggesting that recreational SD alone may provide an insufficiently specific stimulus to induce neuromuscular adaptations comparable to those targeted by structured resistance training.

On the other hand, exploratory analyses revealed favorable within-group changes in selected lipid, hematological, and antioxidant markers, alongside improvements in self-reported health and well-being. Overall, while SD is not a primary strategy for muscular conditioning, it represents a feasible complementary, low- to moderate-intensity option to support active aging, participation, and general well-being within broader public health strategies.

The secondary and exploratory findings require confirmation in prospectively registered studies with adequate statistical power, predefined outcomes, and interaction analyses.

### Limits

This study has some limitations that should be considered. First, the sample size was relatively small. Second, the relatively high attrition rate is a limitation of the present study and may have introduced attrition bias while reducing the statistical power of the analyses. This risk may have been further amplified by the complete-case analysis, which excluded participants without follow-up data. Although withdrawals were mainly due to personal reasons common in older populations (e.g., logistical or scheduling difficulties) and were not attributed to the intervention, the findings should be interpreted with appropriate caution. Another limitation is that habitual PA and dietary adherence were not objectively monitored throughout the follow-up period. Although participants were instructed to maintain their usual lifestyle habits and no major changes were reported, the potential influence of unmeasured variations in Physical Activity or dietary behaviors on the observed outcomes cannot be completely excluded. Finally, since multiple results were analyzed without an overall adjustment, the possibility of Type I errors cannot be ruled out. Secondary and exploratory results should therefore be interpreted with caution, as they are preliminary findings.

Moreover, the trial was not prospectively registered, which represents an additional methodological limitation. Due to the lack of prospective registration of the study, neither a prospective registration identifier nor a registration date is available, and the prespecification of all outcomes and analyses cannot be independently verified. Consequentially retrospective registration would not resolve this limitation.

## Figures and Tables

**Table 1:** Participants’ biometric data at baseline (T0) and after a 6-month intervention phase (T1).

		CG			SDG		
	Time	M	F	Total	M	F	Total
N.		13	12	25	11	13	24
Age (years)		71.5 ± 8.1	68.6 ± 6.2	70.1 ± 7.3	75.8 ± 7.0	73.9 ± 5.8	74.8 ± 6.3
Height (m)		1.68 ± 0.07	1.55 ± 0.06	1.61 ± 0.09	1.67 ± 0.06	1.54 ± 0.06	1.60 ± 0.09
BM (kg)	T0	78.4 ± 12.4	70.3 ± 10.0	74.5 ± 11.9	74.0 ± 7.6	65.7 ± 7.7	69.5 ± 8.6
	T1	79.2 ± 13.6	69.7 ± 9.1	74.7 ± 12.4	73.7 ± 7.8	66.1 ± 8.3	69.6 ± 8.8
BMI (kg/m^2^)	T0	27.7 ± 2.7	29.4 ± 4.3	28.5 ± 3.6	26.4 ± 2.7	27.9 ± 3.4	27.2 ± 3.1
	T1	28.7 ± 4.1	29.2 ± 3.8	28.9 ± 3.9	26.4 ± 2.8	28.1 ± 3.7	27.3 ± 3.4
DBP (mm/Hg)	T0	138.8 ± 13.6	134.1 ± 18.0	136.5 ± 15.7	132.1 ± 9.5	130.9 ± 15.7	131.4 ± 12.9
	T1	129.1 ± 11.3 ***	125.8 ± 14.8 *	127.5 ± 12.9 ***	131.8 ± 11.0	125.0 (120.0–125.0)	127.5 ± 11.0
SBP (mm/Hg)	T0	83.0 ± 7.6	80.9 ± 7.9	82.0 ± 7.6	80.0 (74.0–80.0)	78.9 ± 7.0	80.0 (77.0–80.0)
	T1	77.3 ± 7.4 *	75.0 ± 7.1 *	76.0 (70.0–80.0) ***	80.0 (67.5–80.0)	70.0 (70.0–80.0) *	75.5 (70.0–80.0) *
HR (bpm)	T0	80.7 ± 14.2	76.4 ± 11.7	78.6 ± 13.0	65.6 ± 7.0	70.4 ± 10.6	68.2 ± 9.3
	T1	73.9 ± 8.7 *	71.0 ± 9.1 *	72.5 ± 8.8 **	64.3 ± 7.7	66.5 ± 7.9	65.5 ± 7.7

Abbreviations: BM (body mass), BMI (body mass index), DBP (Diastolic Blood Pressure), SBP (Systolic Blood Pressure), HR (Heart Rate). Non-normally distributed variables are expressed as median (interquartile range), whereas normally distributed variables are expressed as mean ± SD. Paired *t*-test analysis * *p* < 0.05, ** *p* < 0.01, *** *p* < 0.001 vs. T0.

**Table 2:** Participants’ biochemical characteristics at baseline (T0) and after a 6-month intervention phase (T1).

		CG			SDG		
	Time	M	F	Total	M	F	Total
N.		13	12	25	11	13	24
HDL (mg/dL)	T0	44.5 ± 10.9	59.2 ± 13.9	51.6 ± 14.2	45.7 ± 7.0	54.0 (48.0–59.0)	50.5 (44.0–57.0)
	T1	44.0 ± 10.3	59.8 ± 14.7	51.6 ± 14.8	52.5 ± 9.7 **	62.4 ± 13.9 *	53.3 (49.6–63.1) ***
LDL (mg/dL)	T0	93.0 ± 44.0	112.1 ± 41.6	102.2 ± 43.1	98.0 ± 31.3	100.0 (93.0–119.0)	101.0 (90.8–118.3)
	T1	97.0 ± 42.4	100.0 ± 29.8	98.4 ± 36.2	99.5 ± 34.1	97.6 ± 28.4	98.5 ± 30.4
Total Cholesterol (mg/dL)	T0	154.7 ± 51.4	189.3 ± 51.1	171.3 ± 53.2	160.6 ± 37.4	175.5 ± 33.2	168.7 ± 35.2
	T1	163.2 ± 47.8	176.9 ± 35.5	169.8 ± 42.1	165.5 ± 34.2	174.1 ± 32.0	170.2 ± 32.6
Triglycerides (mg/dL)	T0	113.2 ± 45.5	93.0 (78.8–123.5)	111.7 ± 43.2	92.0 (68.5–105.5)	97.5 ± 43.8	89.5 (68.3–105.5)
	T1	87.5 (79.4–154.0)	100.9 ± 35.0	87.5 (65.2–133.0)	87.9 ± 40.1	90.7 (71.7–98.8)	90.9 ± 37.0
Glucose (mg/dL)	T0	93.0 (88.0–102.0)	91.5 (85.3–102.0)	92.0 (87.0–102.0)	91.0 (85.0–96.5)	84.0 (80.0–107.0)	89.0 (82.3–103.3)
	T1	98.4 ± 20.2	89.2 (83.1–96.9)	90.3 (83.7–101.0)	94.1 ± 9.6	87.4 (82.9–94.1)	89.4 (83.7–97.6)
Creatinine (mg/dL)	T0	0.93 (0.84–1.13)	0.82 ± 0.13	0.90 (0.74–0.97)	1.01 (0.78–1.09)	0.75 ± 0.10	0.80 (0.75–0.99)
	T1	0.97 (0.90–1.16)	0.85 ± 0.14	0.90 (0.80–1.05)	0.97 (0.88–1.09)	0.75 ± 0.10	0.82 (0.78–0.94)

Abbreviations: HDL (high-density lipoprotein); LDL (low-density lipoprotein). Non-normally distributed variables are expressed as median (interquartile range), whereas normally distributed variables are expressed as mean ± SD. Paired *t*-test analysis * *p* < 0.05, ** *p* < 0.01, *** *p* < 0.001 vs. T0.

**Table 3:** Participants’ hematological profile at baseline (T0) and after a 6-month intervention phase (T1).

		CG			SDG		
	Time	M	F	Total	M	F	Total
N.		13	12	25	11	13	24
WBCs (×10^3^/µL)	T0 T1	6.39 ± 1.81 6.92 ± 2.01	5.98 ± 1.51 6.59 ± 1.80	6.19 ± 1.65 6.66 (5.02–7.66) *	6.82 ± 1.41 7.00 ± 1.84	5.86 (5.17–6.14) 5.99 ± 1.37	6.05 (5.18–6.94) 6.04 (5.36–7.29)
RBCs (×10^6^/µL)	T0	4.98 ± 0.68	4.69 ± 0.38	4.84 ± 0.56	4.82 ± 0.64	4.59 ± 0.40	4.70 ± 0.52
	T1	5.07 ± 0.63	4.69 ± 0.39	4.89 ± 0.56	5.00 ± 0.80	4.76 ± 0.43 *	4.87 ± 0.62 **
Hb (g/dL)	T0	14.7 ± 1.3	13.8 ± 0.9	14.3 ± 1.2	13.9 ± 1.7	13.3 ± 1.4	13.5 ± 1.5
	T1	14.9 ± 1.2 *	13.7 ± 1.0	14.4 ± 1.2	14.4 ± 2.2	13.7 ± 1.7 **	14.0 ± 1.9 ***
Hct%	T0	45.1 ± 3.9	43.3 ± 3.1	44.2 ± 3.6	42.5 ± 5.2	41.2 ± 4.0	41.8 ± 4.5
	T1	45.4 ± 3.8	42.5 ± 3.3	44.0 ± 3.8	44.5 ± 7.3 *	42.6 ± 4.9	43.4 ± 6.0 **
PLT (×10^3^/uL)	T0	217.6 ± 48.6	184.0 (169.8–265.5)	208.0 (172.0–262.0)	202.1 ± 64.7	238.2 ± 66.1	221.7 ± 66.6
	T1	221.9 ± 36.1	215.0 (172.8–294.8) *	231.0 (182.0–256.0)	176.0 ± 59.2	233.8 ± 71.7	207.3 ± 71.2

Abbreviations: WBCs (White Blood Cells), RBCs (Red Blood Cells), Hb (Hemoglobin), Hct (Hematocrit), PLT (Platelet). Paired *t*-test analysis * *p* < 0.05, ** *p* < 0.01, *** *p* < 0.001 vs. T0.

**Table 4:** Oxidative stress markers and progenitor cell counts.

		CG (n = 25)	SDG (n = 24)
CD34^+^ cells/mL	T0	1.95 (1.42–3.68)	1.81 (1.17–3.00)
	T1	2.73 (1.78–3.83) *	2.21 (1.30–2.98)
	∆%	+47.56	+23.63
BAP (µmol/L)	T0	2154.0 ± 479.0	2223.0 ± 603.0
	T1	2499.0 ± 567.0	2785.0 ± 616.0 ***
	∆%	+19.13	+33.26
d-ROMs (UCARR)	T0	459.3 ± 76.5	458.9 ± 79.5
	T1	458.2 ± 64.8	462.0 ± 64.1
	∆%	−1.28	+1.98

Abbreviations: BAP (Biological Antioxidant Potential); d-ROM (Derivatives of Reactive Oxygen Metabolites). Non-normally distributed variables are expressed as median (interquartile range), whereas normally distributed variables are expressed as mean ± SD. Bonferroni post hoc test; T1 vs. T0, * *p* <0.05, *** *p* < 0.001.

**Table 5:** Participants’ physical fitness and questionnaire results.

		CG (n = 25)	SDG (n = 24)
BMI (kg/m^2^)	T0	28.5 ± 3.6	27.2 ± 3.1
	T1	28.9 ± 3.9	27.3 ± 3.4
	∆%	+1.60	+0.35
TBW (L)	T0	34.9 ± 7.3	34.0 ± 5.8
	T1	34.5 ± 7.2	33.2 ± 6.0
	∆%	−0.99	−2.20
FM (%)	T0	36.8 ± 8.2	36.7 ± 9.1
	T1	37.5 ± 7.9	35.9 ± 9.1
	∆%	+2.14	−1.40
SMM (kg)	T0	26.5 ± 5.6	25.3 ± 4.5
	T1	25.2 ± 5.5 *	25.1 ± 4.5 *
	∆%	−0.96	−0.62
2MST (n. steps)	T0	65.0 ± 19.1	65.7 ± 14.7
	T1	59.8 ± 17.8 *	62.9 ± 21.1 *
	∆%	−7.17	−5.36
PeakT (Nm)	T0	121.9 ± 41.2	117.9 ± 25.9
	T1	115.0 ± 38.0 *	110.7 ± 24.4 *
	∆%	−3.95	−5.70
RTD (Nm/s)	T0	551.9 ± 330.7	492.0 ± 228.4
	T1	450.3 ± 218.4 *	303.9 ± 131.6 *
	∆%	−7.93	−26.4
Fmean (N)	T0	289.7 ± 93.7	302.9 ± 70.6
	T1	283.2 ± 80.2 *	275.3 ± 62.8 *
	∆%	−3.95	−5.70
FI (%)	T0	17.2 ± 8.0	13.2 ± 7.2
	T1	19.7 ± 9.0	15.6 ± 9.0
	∆%	+26.3	+71.3
CST (s)	T0	22.0 ± 4.5	20.8 ± 4.3
	T1	21.0 ± 6.4	20.7 ± 4.0
	∆%	−3.31	+1.64
SP	T0	5.5 ± 1.2	5.8 ± 1.3
	T1	5.6 ± 1.3	5.8 ± 1.4
	∆%	+2.63	+1.09
PASE (leisure and recreational activities score)	T0	12.4 ± 17.8	24.4 ± 22.7
	T1	27.1 ± 36.5 *	49.8 ± 35.0 *
PASE (household activities score)	T0	84.1 ± 36.6	99.3 ± 53.5
	T1	94.6 ± 30.5 *	112.6 ± 40.5 *
PASE (Total score)	T0	96.4 ± 41.4	123.7 ± 64.9
	T1	121.7 ± 50.0 *	162.5 ± 60.9 *
	∆%	+56.9	+79.6
EQ-VAS	T0	75.2 ± 17.9	71.7 ± 17.8
	T1	70.0 ± 19.5	75.0 ± 14.0
	∆%	−5.09	+12.9

Abbreviation: BMI (body mass index); TBW (total body water); FM (fat mass); SMM (skeletal muscle mass); 2MST (2 min Step Test); PeakT (peak torque); RTD (rate of torque development); Fmean (Force Mean); FI % (fatigue index); CST (chair stand test); SP (specific power); Physical Activity Scale for the Elderly (PASE); EuroQol visual analogue scale (EQ-VAS). Paired *t*-test analysis * *p* < 0.05 vs. T0.

## Data Availability

The original contributions presented in this study are included in the article/Supplementary Materials. Further inquiries can be directed to the corresponding author.

## Supplementary Materials

The following supporting information can be downloaded at: https://www.mdpi.com/article/10.3390/jfmk11030330/s1.

## Abbreviations

The following abbreviations are used in this manuscript:

2MST	2 min Step Test
BAP	Biological Antioxidant Potential
BM	Body Mass
BMI	Body Mass Index
CG	Control Group
CST	Chair Stand Test
DBP	Diastolic Blood Pressure
d-ROM	Derivatives of Reactive Oxygen Metabolites
FI %	Fatigue Index
FM	Fat Mass
Fmean	Force Mean
Hb	Hemoglobin
Hct	Hematocrit
HR	Heart Rate
HSPC	Hematopoietic Stem and Progenitor Cells
PeakT	Peak Torque
PLT	Platelet
RBC	Red Blood Cells
RTD	Rate of Tork Development
SBP	Systolic Blood Pressure
SD	Social Dance
SDG	Social Dance Group
SMM	Skeletal Muscle Mass
SP	Specific Power
TBW	Total Body Water
WBC	White Blood Cells
