# Supplementary material for: The Effects of Social Dance on Muscular Fitness and Longevity-Related Circulating Biomarkers in the Elderly
Source: J Funct Morphol Kinesiol. 2026 Aug 24;11(3):330. doi: 10.3390/jfmk11030330 (PMC13608601; doi:10.3390/jfmk11030330)
Supplement: Supplementary file 1 [file jfmk-11-00330-s001.zip › jfmk-4402373-supplementary.pdf]

Table S1. Linear regression model results for the interaction between Gender and Group (Gender × Group) on body composition, physical performance, muscle strength, and quality of life parameters.

Predictor	Estimate	Std Error (SE)	t	*p*-value	CI 95%
**Gender × Group**					
BMI (kg/m2)	-0.21	1.91	-0.110	0.913	[−1.19,1.07]
TBW (L)	2.24	2.59	0.868	0.390	[−0.45,1.14]
FM (%)	-3.17	3.92	-0.808	0.423	[−1.55,0.66]
FFM (kg)	1.64	3.38	0.485	0.184	[−0.58,0.95]
SMM (kg)	0.91	1.89	0.484	0.631	[−0.57,0.93]
CST (s)	-2.74	2.38	-1.151	0.256	[−1.71,0.47]
2MST (n. steps)	-2.11	8.99	-0.235	0.815	[−1.19,0.94]
SP	0.47	0.73	0.647	0.521	[−0.78,1.52]
PeakT (Nm)	32.70	16.3	2.010	0.052	[−0.04,1.91]
Fmean	72.60	39.2	1.850	0.071	[−0.08,1.83]
RTD (Nm/s)	367.0	422.0	0.872	0.388	[−0.62,1.57]
FI (%)	-2.36	4.35	-0.543	0.590	[−1.42,0.81]
PASE (Total score)	47.76	30.10	1.588	0.119	[−0.23,1.95]
EQ-VAS	-4.46	9.82	-0.454	0.652	[−1.36,0.86]

Abbreviation: BMI (Body mass index); TBW (Total Body Water); FM (Fat Mass); SMM (Skeletal muscle mass); 2MST (2-min Step test); SP (Specific Power); PeakT (Peak Torque); Fmean (Force Mean); RTD (Rate of Tork Development); FI % (Fatigue index); CST (Chair Stand Test); Physical Activity Scale for the Elderly (PASE); EuroQol visual analogue scale (EQ-VAS).

Figure S1

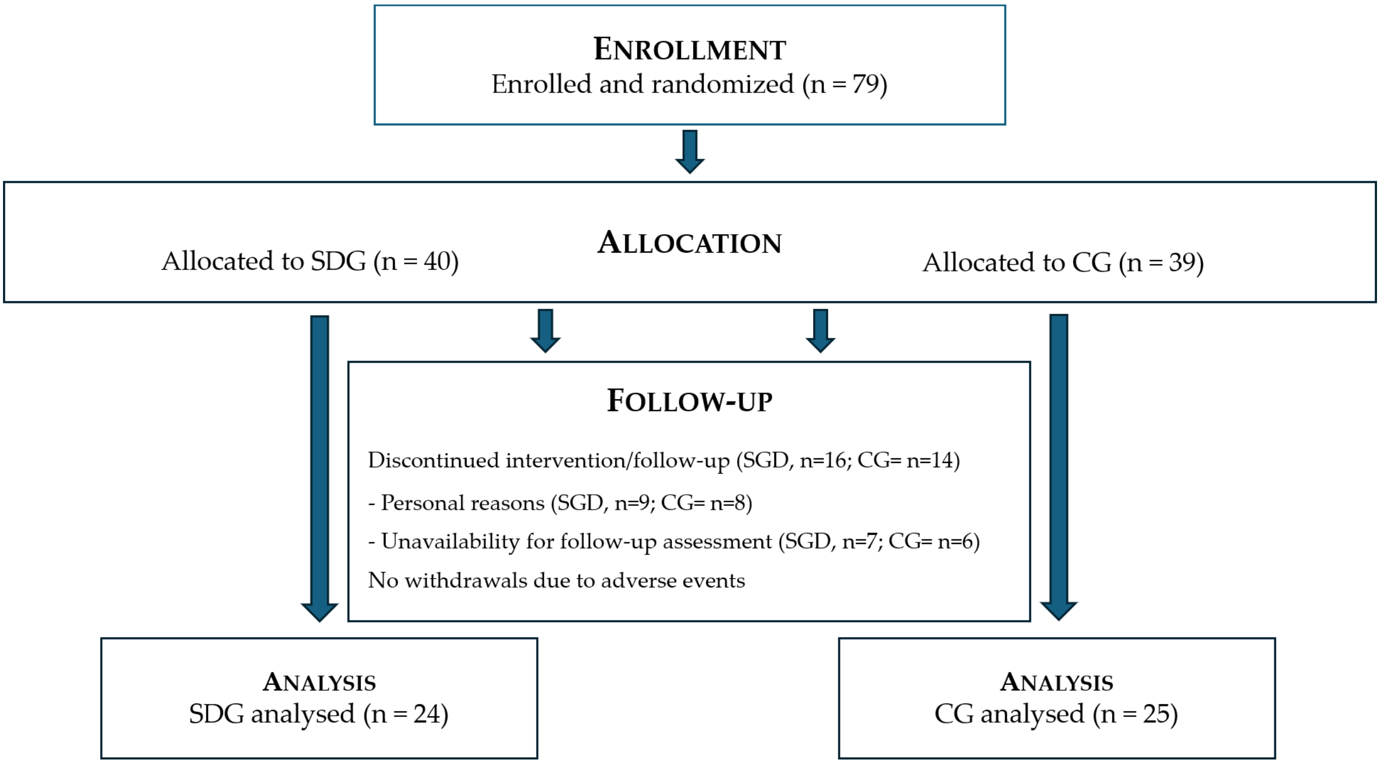

CONSORT Flow Diagram.
